# Infectious Agents as Stimuli of Trained Innate Immunity

**DOI:** 10.3390/ijms19020456

**Published:** 2018-02-03

**Authors:** Paulina Rusek, Mateusz Wala, Magdalena Druszczyńska, Marek Fol

**Affiliations:** 1 Department of Immunology and Infectious Biology, Faculty of Biology and Environmental Protection, University of Lodz, Banacha St. 12/16, 90-237 Lodz, Poland; rusek.paulina93@gmail.com (P.R.); magdalena.druszczynska@biol.uni.lodz.pl (M.D.); 2 Department of Plant Physiology and Biochemistry, Faculty of Biology and Environmental Protection, University of Lodz, Banacha St. 12/16, 90-237 Lodz, Poland; valdek1990@gmail.com

**Keywords:** innate immunity training, epigenetic reprogramming, innate immune memory, bacille Calmette-Guérin (BCG), β-glucan, chitin, lipopolysaccharide (LPS)

## Abstract

The discoveries made over the past few years have modified the current immunological paradigm. It turns out that innate immunity cells can mount some kind of immunological memory, similar to that observed in the acquired immunity and corresponding to the defense mechanisms of lower organisms, which increases their resistance to reinfection. This phenomenon is termed trained innate immunity. It is based on epigenetic changes in innate immune cells (monocytes/macrophages, NK cells) after their stimulation with various infectious or non-infectious agents. Many infectious stimuli, including bacterial or fungal cells and their components (LPS, β-glucan, chitin) as well as viruses or even parasites are considered potent inducers of innate immune memory. Epigenetic cell reprogramming occurring at the heart of the phenomenon may provide a useful basis for designing novel prophylactic and therapeutic strategies to prevent and protect against multiple diseases. In this article, we present the current state of art on trained innate immunity occurring as a result of infectious agent induction. Additionally, we discuss the mechanisms of cell reprogramming and the implications for immune response stimulation/manipulation.

## 1. Introduction

For years, it was consensus that only adaptive immunity has the ability to form a specific kind of immunological memory. Recent reports show that innate immunity forms immunological memory as well. Innate memory occurs as a result of complex regulations, including epigenetic reprogramming in the cell nucleus. Effectors (stimuli) like β-glucan or lipopolysaccharide (LPS) are causative agents in this process. Epigenetic mechanisms entail biochemical and cellular changes, which are manifested by, e.g., enhanced production of proinflammatory cytokines. The mechanism through which “the innate memory” can be mounted is called trained immunity.

When the host organism encounters an infectious agent, its immune response is triggered. This mechanism consists of three steps: recognition of a pathogen, response to its activity (immune reaction), and eradication of pathogen from the host organism [1]. Immunity can be divided into innate (non-specific) and acquired/adaptive (specific) responses—both of them are manifested by cellular and humoral mechanisms. The cellular mechanisms of the innate immunity are characterized by phagocytic and cytotoxic activity of cells, while the humoral response is based on the complement system, lysozyme, and acute phase proteins. During humoral response, cells accumulate at the site of pathogen penetration and secrete molecules (e.g., cytokines), which mediate the inflammatory reaction. The complement system supports phagocytosis, rapidly destroys microbial cells, and controls the inflammatory process. Furthermore, non-specific immunity is combined with specific immunity. Cytokines secreted during the inflammatory reaction condition the type of acquired immunity response, which is connected with the activity of T helper lymphocytes. The cellular mechanism of specific immunity is characterized by the cytotoxic activity of lymphocytes T and their products, namely interleukins (IL) or lymphokines, whereas lymphocyte B products—like immunoglobulins—are responsible for the humoral mechanism [2]. It was established that, besides adaptive immune cells, innate immune cells are also able to mount some kind of specificity through pattern recognition receptors (PRRs), which recognize pathogen-associated molecular patterns (PAMP)–conserved molecular structures produced by microorganisms. It is hypothesized that the capacity for providing immunological memory might be not only the feature of the adaptive immunity but also of the innate immune system [3].

## 2. Trained Innate Immunity—General Characteristics and Mechanisms

Training of the innate immunity is based on non-specific immunity stimulation by bacterial-, fungal-, or viral-derived particles. Although induced immune response is effective against a certain range of pathogens or PAMP molecules, it does not have a specific character depending on a particular infectant/stimulant. For example, during training of human monocytes with β-glucan (a component of the fungal cell wall) from *Candida albicans* (a human opportunistic pathogen), the immunity is induced not only against fungi, but also against bacteria, viruses and even parasites [4]. Furthermore, it was observed that training of human monocytes induced by chitin from *Saccharomyces cerevisiae* (another human opportunistic pathogen) leads to enhanced capacity to eliminate microbes like *Candida albicans*, *Staphylococcus aureus* (Gram-positive bacteria), or *Escherichia coli* (Gram-negative bacteria) compared to non-trained human monocytes [5].

**Figure 1:**
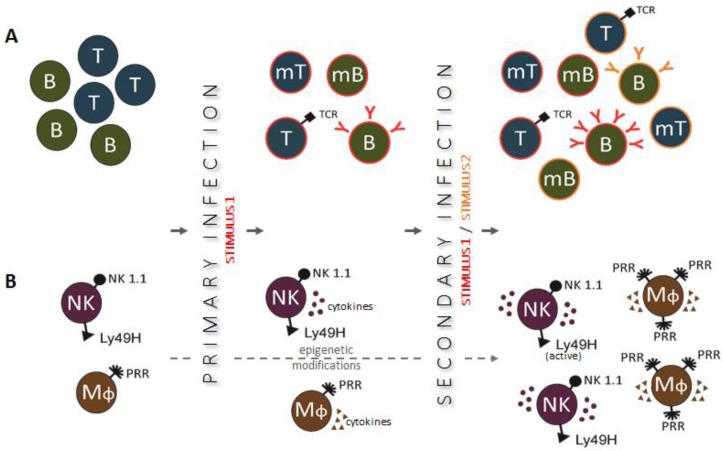
Primary and secondary immune responses in adaptive (panel **A**) and innate (panel **B**) immunity. (**A**) After recognition of an infectious agent, naïve T and B cells transform into antigen-specific effector cells, which can survive as memory cells and respond more robustly to the same infectious agent during secondary infection; (**B**) Innate immune cells, activated during primary infection, undergo epigenetic reprogramming and become primed to respond more effectively to secondary stimulation caused by a related or unrelated infectious agent.

Unlike non-specific immunity, specific immunity has the ability to produce immunological memory cells, and, as a consequence, the precise immunological defense is promptly launched during reinfection by the same pathogen. It is hypothesized that trained innate immunity could have an adaptive character (Figure 1) and could also mount some kind of memory. These processes happen independently of T and B cells [6]. The phenomenon of trained innate immunity is accompanied by epigenetic mechanisms involving histone modifications and reconfiguration of chromatin, as well as it is most likely related to DNA methylation or modulation of microRNA and/or long noncoding RNA expression. This results in the reprogramming of the monocyte, macrophage, or natural killer (NK) cell function, expressed as the alteration in intracellular immune signaling and remodeling of cellular metabolism from oxidative phosphorylation toward aerobic glycolysis. This way, the innate immune cells are able “to acquire” enhanced capability to respond to certain stimuli [4].

It is well known that after being recognized by PRRs, microbial ligands exert different effects on the functional program of immune cells. The changes are connected with an epigenetic profiling mediated especially through histone modifications, which affect cytokine production and immune protection against nonrelated infections. Modifications on the histone tail are added or removed by enzymes, so-called writers and erasers, respectively. The modifications include e.g., methylation, acetylation, or phosphorylation [7]. Acetylation is associated with the activation of gene transcription, whereas methylation—which is connected with condensation of chromatin—prevents binding of transcription factors and gene silencing. However, the final effect of epigenetic cell reprogramming connected with histone methylation depends in a large measure on the position of methylated amino acid residues and the number of bound methyl groups. The activation of gene transcription is often linked to the methylation of Histone 3, Lysine 4 (H3K4), H3K36, and H3H79, while the repression of gene transcription is a result of H3K9, H3K27, and H4K20 methylation [8,9]. Histone modifications determining chromatin compaction affect the access of transcription factors to specific DNA sequences. The effects of transcription factor binding and its consequences for differentiation, tolerance, and training have been reviewed in detail elsewhere [7,10]. One of the most studied epigenetic modifications in the context of the innate immunity training is the methylation process of Histone 3 (H3), and especially trimethylation of the fourth amino acid residue (lysine) from the N-terminus (H3K4me3) [11]. The increased level of H3K4me3 in monocytes after β-glucan treatment was detected mostly in regions associated with the promoters of genes responsible for proinflammatory cytokines expression, like tumor necrosis factor (TNF) α or interleukin (IL) 6 and IL-18 [12,13]. LPS-stimulated dendritic cells increase the level of H3K4me3, which remains stable up to two hours after stimulation [14]. A similar phenomenon is observed in macrophages—after stimulation, the levels of H3K4me3 and H3K4me increase in the regions of promoters and enhancers of numerous genes. After a few hours, most of H3K4me3 returns to the initial level [15,16]. On the other hand, H3K9me is related to transcription repression. After cell stimulation with LPS, the repression is abolished and transcription of genes coding proteins, like IL-12b, is induced [17]. Dendritic cells, which are professional interferon (IFN) producing cells, demonstrate a lower level of H3K9me2 compared to fibroblasts or heart myocytes, which produce an inconsiderable quantity of IFN. Furthermore, genetic abolishment of H3K9 methyltransferase G9a leads to increased IFN production in fibroblasts and makes virus suppression possible [18].

Acetylation is a posttranslational reversible histone modification catalyzed by histone acetyltransferases (HATs), which transfers the acetyl residue from acetyl-CoA to lysine residues. Histone deacetylases reverse this process. Macrophages stimulated with LPS demonstrate an enhanced level of acetylation at histone 4 (H4Ac), which results in chromatin opening. Acetylation of lysine 5, 8, and 12 on a histone occurs in the regions connected with selected promoters up to one hour after stimulation and decreases after two hours. In dendritic cells, the level of H4K29Ac grows dynamically during LPS stimulation [7].

As a result of studies conducted over the past few years, epigenetic regulations of immunological processes have gained increasing attention. Along with those discoveries, the concept of innate immunological memory is rapidly developing. That kind of memory is formed after stimulation of innate immunological cells with microorganisms. There are many expectations related to the possibility of using the phenomenon of trained innate immunity (and its epigenetic regulation) in the treatment of diseases connected with immunodeficiency, for example, for which there is no effective therapy. However, it should be mentioned that epigenetic reprogramming may also contribute to tumorigenesis [19]. A good example of that is the influence of gut microbiota on human epithelial cells, whose proliferation, differentiation, and secretory activity can be affected by a vast variety of bacterial low molecular weight compounds. They modulate signaling pathways and regulate gene expression through, e.g., DNA methylation, posttranslational modification of histone proteins and genomic imprinting as well as act indirectly by inhibition of certain enzymes such as DNA methyltransferases (DNMTs), histone deacetylases (HDACs), and telomerase reverse transcriptases (hTERTs) [20,21,22]. As reviewed by Paul et al. [21] the gut microbiome may play a role in the development of intestinal environmental changes leading to progression toward gastric and colon cancer, hormone-dependent, endometrial, ovarian and breast cancer, or liver and lung cancer. Nevertheless, there is some evidence that the gut microbiome is a critical player in the regulatory responses. It is believed that gastrointestinal flora creates conditions favoring tollerogenic Foxp3^+^ regulatory T (T_reg_) cell development. Thus, the tolerance to commensal and environmental antigens is determined by T_reg_ cells delivered during thymic development as a result of classic self/non-self discrimination mechanisms as well as by T_reg_ cells appearing extrathymically at mucosal sites [23,24,25].

## 3. Biochemical and Cellular Mechanisms of Training

**Figure 2:**
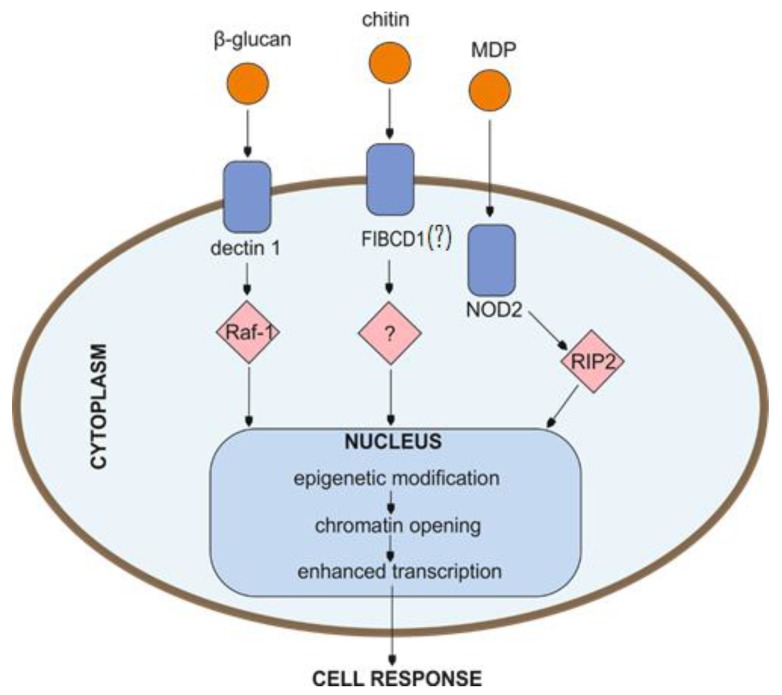
Potential cell signal-transduction pathways involved in the induction of trained innate immunity. Signaling pathways triggered in the response to certain stimuli lead to cell functional reprogramming associated with epigenetic changes resulting in the regulation of the ensuing cell phenotype. MDP—muramyl dipeptide; NOD2—nucleotide binding oligomerization domain containing 2; Raf-1—Raf-1 proto-oncogene, serine/threonine kinase; RIP2—receptor-interacting protein kinase; FIBCD1—fibrinogen C domain containing 1 protein (although mainly expressed on enterocytes and airway epithelial cells, due to the high homology to the ficolins, it may play an important role in innate immunity).

Stimulated innate immune cells manifest nonspecific features, evoking certain characteristics of adaptive immunity, described also in plants and invertebrates. Research indicates that during training those cells show an enhanced response to factors produced by pathogenic microbes. Some of the most commonly studied stimuli in the aspect of trained innate immunity are β glucan, chitin, BCG vaccine, or LPS (Figure 2). Interestingly, the repertoire of training inducers is not only of a chemical nature, but it can be extended by physical activity. Studies on horses exhibited that moderate exercises resulted in the alteration of mRNA expression for certain TLRs and selected cytokine secretion by blood monocytes and pulmonary alveolar macrophages treated in vitro with different TLR ligands [26].

### 3.1. β-Glucan

*Candida albicans*, which is a part of healthy human mycobiota, inhabits the oral cavity, gastrointestinal tract, and vagina. This commensal microorganism is transmitted from mother to infant during birth, which initiates a life-long coexistence between the fungus and its host. Under certain conditions this microorganism is able to lead to the development of disease, thus the elucidation of the influence of fungus components on the immune cells is a pivotal matter for the understanding of the host–microbe interplay [27,28]. Studies on the effect of the initial treatment of wild strain mice with *Candida albicans* have been used to estimate the influence of monocyte training on proinflammatory cytokine production. Cytokine production induced by LPS was enhanced, when mice were treated one week earlier with a low dose of *Candida albicans* cells killed with high temperature. The main group of cells responsible for proinflammatory cytokine production during pathogen infection are peripheral blood mononuclear cells. It was observed that in vitro treatment (priming) of PBMCs with a low dose of *Candida albicans* induced response of the cells after their secondary stimulation (restimulation) with the same microorganism, and what is particularly interesting—also with different PRR ligands and even other bacteria. Although both β-glucan and mannan are the most abundant components of the *Candida albicans* cell wall, only the first one showns the same abilities as the whole cells of *Candida albicans* do. The most effective stimulation was noted after 24 h of preincubation and was expressed through enhanced production of TNF-α and IL-6 but not anti-inflammatory IL-10. Furthermore, the cells were able to sustain the increased production of cytokines up to two weeks after the training period. The training was not effective in the case of NK cells (CD56+), whereas monocytes responded additionally with the enhanced candidacidal activity. It was reported that the dectin-1/Raf-1 signaling pathway is required for the training induction, and the underlying epigenetic modifications of the training are associated with the increase in H3K4me3 at the level of the promoter of genes whose products (e.g., Myd88, Raf-1) are involved in the immune signaling pathways and the training mechanism [12]. It has been reported that signal transducer and activator of transcription (STAT)-1 is involved in Candida-induced trained immunity. This observation relies on the results of in vitro experiments, in which the innate immune cells were isolated from patients suffering from chronic mucocutaneous candidiasis (CMC) or hyper-immunoglobulin (Ig) E syndrome (HIES). Both primary immunodeficiencies are characterized by a defect in T helper type 17 (Th17) cytokine production capacity and are a result of mutations in STAT1 and STAT3 genes, respectively. While CMC patients are susceptible mainly to Candida and dermatophyte infections, HIES patients suffer particularly often from skin and respiratory tract infections caused by *Staphylococcus aureus* and *Streptococcus pneumoniae*. It has been shown that STAT-1 is crucial for immune training with *Candida albicans* and is accompanied by an IFN-γ-dependent mechanism [29]. It has also been found that numerous modifications of histone (H3K4me2, H3K4me3, H3K9me3, and H3K36me3) are correlated with STAT-1 activation by IFN-γ [30]. It is noteworthy that although the training of PBMCs from HIES patients, with the use of either whole cells of *Candida albicans* or β-glucan, was effective, it failed in the case of CMC patients when *Candida albicans*, but not β-glucan, was used. It might suggest that other components of the *Candida albicans* cell wall or other cell wall-associated particles can be involved in innate immune training [29]. Garcia-Valtanen et al. [31] observed that β-glucan-primed monocytes in vitro were able to differentiate into the cells morphologically corresponding to macrophages, however the levels of certain surface molecules (e.g., F4/80, CD11b, CD11c, MHC II) decreased compared to control macrophages. These cells responded with the high production of TNF-α and IL-6 after reincubation with LPS as the second stimulant, which at least partially could be explained by the enhanced cell survival resulting from the reduction of cell apoptosis by β-glucan. Furthermore, the enhanced cytokine production was also observed in vivo in a mouse model. However, the effect of β-glucan training followed by LPS challenging has waned in time, which suggests a transitory character of β-glucan-induced trained immunity [31]. Understanding the immunological background of the innate immune memory development as well as the potential role of different molecules engaged in the course of this phenomenon can provide an impulse for designing new, innovative immunotherapeutic approaches [32].

### 3.2. Chitin

Besides Candida spp., *Saccharomyces cerevisiae* is also found on the human skin and in the intestinal tract. Since both of the fungi colonizers share many cell wall structures, it is possible that they may similarly influence the host immunity. Different strains of *Saccharomyces cerevisiae* as well as *Saccharomyces cerevisiae*-derived chitin were used by Rizzetto et al. [5] to induce innate immunity training in monocytes in the context of the enhancement of their killing ability. Chitin, similarly to β-glucan is a primary component of the fungal cell wall. Although it has been shown that chitin is recognized by FIBCD1 (Fibrinogen-like recognition domain containing 1), it is still not known how chitin-triggered signal is transduced to the nucleus [33]. After primary stimulation, the monocytes were exposed to live *Candida albicans*, *Staphylococcus aureus*, or *Escherichia coli* for six hours, and after that time they exhibited an enhanced ability to kill the pathogens in contrast to non-stimulated control cells. A particularly strong effect was observed in monocytes trained with chitin. Thus, it was experimentally verified that chitin from the fungal cell wall can train innate immunity. Trained monocytes were found to intensify TNF-α and IL-6 production during the second contact with the pathogen. It was found that the training was restrained by the use of the inhibitor of histone methyltransferases, that suggests the role of epigenetic changes at the level of histone methylation in reprogramming of chitin-trained monocytes [5].

### 3.3. Lipopolysaccharide (LPS)

The pattern of exposure to stimuli may determine either the enhancement state (trained immunity) or the tolerance state. The final effect is tightly connected with the nature of the first challenge. One of the most intensively described agents in this context is LPS (known as endotoxin), which is a major component of the cell wall in most Gram-negative bacteria [34]. This antigen is recognized by TLR4. It has been reported that the effect of LPS stimulation on inflammatory response is dose-dependent. A primary contact of monocytes and macrophages with high doses of LPS drives them into a tolerance state, whereas low LPS concentrations prime the cells towards an enhanced immune response to the secondary stimulus. High dose LPS-derived tolerance state allows avoiding an excessive immune response during the secondary stimulation, which could lead to tissue damage. On the other hand, the enhanced responsiveness to low LPS doses induces innate training, which results in an enhanced reactivity to subsequent stimulation. Nevertheless, both states are mediated through similar histone modifications, but by a different pattern of gene expression [3,11] The dual nature of LPS-derived training is so far mostly unknown and is probably associated with the dose, timing, and type of the subsequently used immune response inducer [35].

### 3.4. Bacille Calmette-Guérin (BCG)

Early studies indicated that BCG vaccine immunization could induce non-specific, protective effect against other pathogens. Mice vaccinated against tuberculosis appeared to be protected also when secondary infections with e.g., *Staphylococcus aureus*, *Listeria monocytogenes*, *Salmonella typhimurium*, or *Schistosoma mansoni* occurred. This non-specific protective effect was observed even in mice deficient in T and B lymphocytes. This allowed supposing that the key role in the observed phenomenon could be played by monocytes/macrophages [36,37,38,39,40]. However, mononuclear phagocytes are not the only cells participating in the BCG-derived nonspecific protection against reinfection. The BCG vaccine has also a crucial influence on NK cells. Fractionated NK cells from vaccinated healthy volunteers showed enhanced proinflammatory cytokine production as a response to secondary pathogenic bacteria and fungi infection. Furthermore, it is known that NK cells can recognize BCG without the engagement of antigen-presenting cells [41]. Enhanced human NK cells and monocytes response, stimulated with the BCG vaccine, is caused by epigenetic modification at the H3K4me3 level. This process is mediated through the signal transduction pathway, in which NOD2 protein is an intracellular pattern recognition receptor [42]. As it was recently shown by Kleinnijenhius et al. [43], the NK cells isolated from BCG vaccinated volunteers responded with the enhanced production of IL-1β, IL-6, TNF-α, but not IFN-γ after their ex vivo stimulation with mycobacteria-related or unrelated antigens. It was also reported that NK cells are at least partially responsible for the increased survival of BCG vaccinated SCID mice which were infected with a lethal dose of *Candida albicans*. Experiments on mice indicated that macrophages, stimulated with *Mycobacterium bovis* BCG, were able to enhance hydrogen peroxide (H_2_O_2_) production. It was observed that after vaccination, mice lacking functional lymphocytes demonstrated enhanced survival in sepsis caused by Candida. In this case, splenic monocytes produced higher levels of TNF after ex vivo stimulation. Moreover, BCG vaccinated mice, compared to non-vaccinated individuals, were more effectively protected against malaria. It is suggested that it was attributable to intensified transcription of antibacterial proteins [44]. Interestingly, BCG training, unlike the β-glucan one, induced only minimal changes in morphology of monocytes. Microscopy observations indicated that the cell size increased slightly to 29.2 µm from 20.6 µm of untrained control cells. The restimulation with LPS or synthetic lipopeptide Pam3Cys caused no further size changes. After restimulation, the trained cells responded with the elevated production of proinflammatory cytokines. Although the training with BCG induced a less spectacular increase in cytokine production by monocytes upon their restimulation than the training with β-glucan did, still the IL-6 and TNF-α production was 4–5-fold higher compared to the control cells. Moreover, it was indicated that the BCG training did not lead to an increase in cell numbers in the in vitro culture, hence the increase in cytokine levels was due to the amount of cytokines produced by each cell. Furthermore, training with BCG resulted in an increased production of reactive oxygen species (ROS) by monocytes after their stimulation with opsonized zymosan, whereas β-glucan trained cells were characterized by downregulation of ROS production [45]. Interesting data came from the study on human monocytes isolated from volunteers two weeks, three months, and one year after their immunization with BCG [43]. It was shown that monocytes obtained from immunized individuals responded with a significantly enhanced TNF-α and IL-1β production after stimulation with sonicated *Mycobacterium tuberculosis* H37Rv or with non-related pathogens, such as heat-killed *Candida albicans* and *Staphylococcus aureus*. This effect was observed as long as three months after vaccination. What is interesting is that the cytokine production induced by LPS was significantly enhanced even one year after BCG vaccination in contrast to the production before vaccination. Furthermore, one year post-vaccination monocytes were characterized by an enhanced expression of CD14, CD11b, TLR4, and the mannose receptors, while the expression of TLR2 or dectin-1 remained unchanged. All of the above suggest that the beneficial effect of BCG vaccination could be the induction of the innate immunity reprogramming expressed as the long-term sustained changes in the non-specific resistance against infections [46].

### 3.5. Plasmodium falciparum

A state of trained innate immunity could be also induced by *Plasmodium falciparum*, the causative agent of malaria. Studies with the use of *Anopheles gambiae* mosquitoes suggest that the primary *Plasmodium falciparum* infection primes the mounting of innate immune memory that partially protects against a secondary infection with Plasmodia [47]. Using an experimental malaria model, McCall et al. [48] demonstrated that *Plasmodium falciparum* re-routed human TLR responses toward a more proinflammatory cytokine profile both in vivo and in vitro. Malaria-induced IFN-γ-dependent priming was suggested to induce a trained immunity component [49,50]. Luty et al. [49] showed that Gabonese children suffering from malaria, whose peripheral blood mononuclear cells produced IFN-γ in response to *Plasmodium falciparium* peptides, had significantly lower rates of malaria reinfections than children, whose PBMC did not secrete this cytokine after the parasite stimulation. Similarly, Dodoo et al. [50] demonstrated that production of malaria-specific IFN-γ by PBMC from Ghanaian malaria patients was associated with a reduced risk of malarial disease and reinfection.

### 3.6. Hepatitis B

Hepatitis B virus (HBV), a cause of liver inflammation and cancer in chronically infected individuals, is also able to trigger a state of trained immunity. The risk of developing chronic HBV infection varies with age and is the highest in children born to HBV-infected mothers compared to individuals infected later during childhood or adulthood [51]. It has been demonstrated that HBV exposure in utero induces in neonates a trained immunity profile, which enhances the ability of their immune cells to respond to bacterial infections in vitro [52]. Such effect has been found to be a result of increased innate immune cell maturation and Th1 development as well as alterations in the cytokine profile. Cord blood mononuclear cells from neonates born to HBV+ mothers produced higher concentrations of IL-12p40 and IFN-α2 in response to unrelated bacterial pathogens (*Pseudomonas aeruginosa*, uropathogenic *Escherichia coli*, *Salmonella typhimurium*, *Acinetobacter baumanii*, *Listeria monocytogenes*) compared with healthy controls. The levels of IL-10 and pro-inflammatory cytokines such as IL-6, IL-8, and TNF-α were significantly lower in bacterial-stimulated cord blood cultures from HBV-exposed children than in healthy individuals. The induction of trained immunity in HBV-exposed neonates suggests that the neonatal immune system can be trained by the virus allowing protection from unrelated microbial pathogens during early life [52].

## 4. Significance of the Trained Innate Immunity Phenomenon

The observation that the innate immune cells can “preserve memory” regarding the primary stimulus and show heightened responsiveness to subsequent stimulation could be an inspiration for the elucidation of the mechanisms of the development of certain diseases and for designing of innovative therapeutic solutions.

Beneficial effects of the BCG vaccine, that go beyond its primary anti-tuberculosis feature, have already been observed previously. A reduced mortality level was recorded among vaccinated newborns with low birth weight in contrast to unvaccinated newborns. It was also suggested that the exposure to the microorganism and its products in the early life stage may reduce asthma risk in adult life [53]. Many studies indicate that a state of trained immunity occurs early in life, after birth. Human neonatal cord blood mononuclear cells after in vitro stimulation with *Staphylococcus epidermidis* showed enhanced expression of various pattern recognition receptors (PRR) including Toll-like receptors (TLR) allowing greater innate immune response to nonspecific microbial infections [54]. A newborn murine model confirmed upregulation of TLR2 mRNA after intravenous infection with *Staphylococcus epidermidis* [55].

Experiments on mice lacking specific immunity showed that after BCG vaccination the protection from *Candida albicans* infections, to which they were susceptible, was induced. It was concluded that this effect resulted from the induction of trained innate immunity triggered by BCG vaccine stimulation. Moreover, recent studies indicate that human monocytes and NK cells exhibit an enhanced reaction during restimulation [42].

It is suggested that non-specific effects of BCG vaccination can be used in enhancing the effects of low-efficient vaccines, for example the *Salmonella typhi* vaccine or the influenza vaccine. The BCG vaccine may play a crucial role in immunodeficiency patients, for example HIV infected ones. On the other hand, considering the fact that the BCG vaccine is a live vaccine, the possibility of using it as a stimulant in innate immunity training is restricted in that group [41]. Many non-specific effects of other well-known vaccines are also possible, as it was reviewed by Blok et al. [44]. Those effects may be caused by innate immunity reprogramming. Therefore, vaccines, like those against smallpox or measles, which may demonstrate non-specific effects, should be studied. Recognition of those non-specific effects may lead to the development of therapies, in which vaccines can be used as an element of the treatment of emerging recalcitrant disorders [44]. To date, it has been observed that the beneficial non-specific effects related to the decrease in mortality and morbidity or even reduction in melanoma risk were associated with the live vaccine use [56,57,58,59,60], while the immunization with inactivated combined whole-cell diphtheria–tetanus–pertussis (DTP) vaccine resulted in higher mortality for DTP-vaccinated compared with DTP-unvaccinated individuals [61]. Moreover, depending on the sex of the vaccinated patients, there were different effects noticed—BCG vaccinated newborn males showed a beneficial effect sooner than newborn females. On the other hand, the measles vaccine showed more beneficial effects in females than in males. Interestingly, antibodies of BCG-vaccinated mothers had an influence on infants—an enhanced protection against the acute phase of diseases was noticed [62].

The phenomenon of trained innate immunity could be applied to help the cancer patients suffering from infections. It has been shown that some cancers are able to induce an immunosuppressive environment through the production of anti-inflammatory cytokines (e.g., IL-10, TGF-β, and indoleamine 2,3-dioxygenase) altering the balance from a Th1/Th17 towards a regulatory (FOXP3) T cell phenotype and due to the expression of certain immunosuppressive ligands (programmed death-ligand 1 (PD-L1) and 2 (PD-L2) being responsible for the inhibition of effector memory T cell proliferation and activation [63]. The beneficial effects of BCG vaccination have been reported for decades. The reports that were published as soon as a few years after the introduction of the BCG vaccine indicated that the dramatic drop in mortality observed among BCG vaccinated children could not only be the result of antimycobacterial properties of the vaccine [44]. Over time, the BCG vaccine has been successfully used as the supportive treatment of urothelial cell carcinomas and melanomas. Moreover, the BCG beneficial effect is suggested also in the case of malignant disease. In the view of the present knowledge, the BCG-derived beneficial results could be explained by the phenomenon of the innate immune cell reprogramming. BCG-induced non-specific immune memory attributed to monocytes and NK cells provides the potential for more effective protection against infectious agents different from the primary ones and allows reversing the immunosuppressive tumor microenvironment [63]. As it was listed by Tӧpfer et al. [11], while planning the use of vaccine formulations to achieve the non-specific immune effects through the innate immune training the following factors should be taken into account: the sex, ethnicity, and age of the recipients; sequence/timing and combination of vaccines; the conditions of the vaccine production process (e.g., microorganism growth rate); or the properties of the innate immunity reprogramming by adjuvants that are already in use.

## 5. Conclusions

The phenomenon termed “trained innate immunity” or “innate immune memory” complements or even rebuilds our understanding of the immune system functioning. So far, the innate immunity has been considered to be more primitive and less sophisticated compared to the adaptive one. However, the described capability of the innate immune cells to mount immunological memory after the primary stimulation leading to a heightened non-specific response after their re-stimulation with the same or different stimuli suggests that the innate immunity is more complex than it has been thought. The epigenetic changes/modifications are at the heart of the cell reprogramming and result in rewiring of gene transcription. The main attention is focused on monocytes and macrophages, nevertheless NK cells undergo training as well. This feature can exert beneficial effects on the host, e.g., by the induction of protection from infectious agents other than the primary stimulus or by the reversion of some processes accompanying tumor formation.

The durable effect of innate immune training could be explained at least partially by the epigenetic reprogramming at the level of progenitor cells, which seem to be able to transmit their phenotype down their lineage [64,65]. For instance, the studies in a mouse model showed, that bone marrow-derived dendritic cells (BMDC) obtained from animals colonized with segmented filamentous bacteria (SFB) exhibited protective properties against *Entamoeba hystolytica* due the elevated production of IL23 and IL-17A. What is most interesting, when transferred to SFB noncolonized mice, those BMDC preserved their enhanced cytokine production and protective anti-*Entamoeba hystolytica* function [66].

Better characterization of trained immunity would provide “epigenetic tools/drugs” that, used separately or in combination with traditional therapies, will offer more effective disease treatment. Further studies will probably bring many answers and allow a better understanding of interactions between non-specific and specific immunity.
